# Knotless PEEK and double-loaded biodegradable suture anchors ensure comparable clinical outcomes in the arthroscopic treatment of traumatic anterior shoulder instability: a prospective randomized study

**DOI:** 10.1007/s00167-022-06969-6

**Published:** 2022-04-18

**Authors:** Maristella F. Saccomanno, Simone Cerciello, Marco Adriani, Marcello Motta, Almerico Megaro, Stefano Galli, Alessandra Scaini, Giuseppe Milano

**Affiliations:** 1 Department of Medical and Surgical Specialties, Radiological Sciences and Public Health, University of Brescia, Brescia, Italy; 2 Department of Bone and Joint Surgery, Spedali Civili, Brescia, Italy; 3 Department of Orthopaedics, Fondazione Policlinico Universitario Agostino Gemelli, Rome, Italy; 4 Ortopedia e Traumatologia 2, Spedali Civili, Largo Spedali Civili 1, 25123 Brescia, BS Italy

**Keywords:** Shoulder instability, Arthroscopy, Bankart repair, Suture anchor, Biodegradable, PEEK

## Abstract

**Purpose:**

To compare the clinical outcome of arthroscopic capsulolabral repair for traumatic anterior shoulder instability with PEEK knotless and knotted biodegradable suture anchors.

**Methods:**

Arthroscopic stabilization was performed in 78 patients with recurrent traumatic anterior shoulder instability. They were divided into 2 groups of 39 patients each, according to suture anchors used: knotless PEEK anchors in group 1, and biodegradable anchors in group 2. Exclusion criteria were: instability without dislocation, posterior or multidirectional instability, glenoid bone loss > 20%, off-track lesions, concomitant rotator cuff tears and previous surgery. The primary outcome was the Disabilities of the Arm, Shoulder and Hand (DASH) self-administered questionnaire. Secondary outcomes were: Work-DASH, Sport-DASH, Rowe score, recurrent instability and subsequent surgery. The following independent variables were considered: age, gender, dominance, generalized ligamentous hyperlaxity, duration of symptoms, age at first dislocation, number of dislocations, type of work, type of sport, sports activity level, capsule-labral injury pattern, SLAP lesion and number of anchors. Differences between groups for numerical variables were analyzed by use of the Student’s *t*-test or Mann–Whitney *U*-test. Fisher’s exact test was used for analysis of categorical variables. Significance was set at *p* < 0.05.

**Results:**

Seven patients (9%) were lost at follow-up, 5 from group 1 and 2 from group 2. Follow-up ranged from 36 to 60 months (median: 44; IQR: 13). Comparison between groups did not show significant differences for each independent variable considered. No differences could be found either for DASH (n.s.) or Rowe (*p* = n.s.) scores between the two groups. Overall recurrence rate was 7%. Three re-dislocations were reported in group 1 and two in group 2 (n.s.). Only one patient in each group underwent re-operation.

**Conclusions:**

The study showed no significant differences in clinical outcomes after arthroscopic treatment of traumatic anterior shoulder instability using PEEK knotless or biodegradable knotted anchors at mid-term follow-up.

**Level of evidence:**

I.

## Introduction

Suture anchors represent the gold standard for arthroscopic Bankart repair. Historically, metal anchors were the first to be introduced, albeit their use for labral repair was rather discontinued and absorbable suture anchors were popularized [21]. Major strengths of using biodegradable anchors rely on their compatibility with any imaging technique and theoretically on the opportunity of easier revision surgery. Several clinical trials compared clinical outcomes of arthroscopic Bankart repair with metal and absorbable suture anchors showing no significant differences between them [19, 25]. An alternative to metal or biodegradable materials is polyether-ether-ketone (PEEK). PEEK is a stable, inert material resistant to hydrolysis and oxidation with no evidence of cytotoxicity [11].

Anchor design may be important for safety and efficacy of tissue-to-bone fixation. Nowadays, knotted or knotless anchors are available. Potential major drawback of knotted anchors depends on the presence of bulky knots protruding over the articular surface, which can cause friction and cartilage wear. Moreover, arthroscopic knot tying is time-consuming and often requires a certain learning curve. On the opposite, knotless anchors have the advantage of being easy and fast to use.

Theoretically, safe, reliable and effective fixation devices for arthroscopic Bankart repair should consist of a combination of inert material with the most suitable design. Although several ex-vivo studies compared mechanical behavior of different suture anchors available on the market [1, 14, 23], few clinical trials compared safety and efficacy of suture anchors having different composition and design [4, 18, 22].

The purpose of the present study was to compare the clinical outcome of arthroscopic capsule-labral repair for anterior shoulder instability with PEEK knotless and knotted biodegradable suture anchors. The hypothesis of the study was that there were no differences in clinical outcome between the two types of suture anchors.

## Materials and methods

The protocol and informed consent process were approved by the institutional review board and by the local ethic committee (prot no. 763A1411, Catholic University, Rome). A single-blind prospective randomized controlled trial was conducted.

### Population

Patients aged 18 years or older affected by recurrent traumatic anteroinferior glenohumeral instability who accepted to enter the study were considered eligible. All patients underwent a bilateral computed tomography (CT) scan preoperatively to rule out critical bone defects. Exclusion criteria were: instability without dislocation, posterior/multidirectional instability, glenoid bone defect exceeding 20% of the surface area of the inferior glenoid [26], off-track lesions [9], Bony Bankart lesions, concomitant rotator cuff tears, previous fractures and/or surgery on the same shoulder, infections, congenital or acquired inflammatory or neurologic diseases (systemic or local) involving the shoulder girdles, inability or unwillingness to sign the informed-consent form or to complete self-administered questionnaires. Enrollment was confirmed at the time of surgery.

### Randomization and allocation concealment

Patients were divided into 2 groups comprising 39 cases each and were randomly allocated with a random sequence generator (www.random.org). The randomization list was kept by an independent researcher not involved in the study. Allocation concealment was performed by use of a closed-envelope procedure, and the assignment code of each patient to one of the two groups was shown to the surgeon only at the time of surgery, after confirmation of enrollment.

### Intervention

All patients underwent an arthroscopic anterior capsulolabral repair. Groups differed in the type of suture anchors used: 2.9-mm PEEK knotless anchors (Pushlock 2.9 mm; Arthrex Inc., Naples, FL, USA) in group 1; and double-loaded bioabsorbable anchors consisting of amorphous poly-l/d-lactic acid (PLDLA) (Bio-FASTak 3.0 mm; Arthrex) in group 2.

In all patients, surgery was performed in general anesthesia and in lateral decubitus position. A standard three-portals surgical approach was used. Capsule-labral status was documented according to the injury pattern of the anterior-inferior capsule-labral complex (Bankart or ALPSA lesion) and the presence and type of SLAP lesion.

Concomitant SLAP lesions were repaired. All the operations were performed by the same surgeon. After surgery, a sling was applied to the operated limb and maintained for 4 weeks; after this period, all patients underwent the same rehabilitation program for at least 2 months. In the first phase (4–8 weeks), rehabilitation was focused on regaining a full range of motion; while in the second phase (9–12 weeks), patients underwent a muscle-strengthening program. Return to work or sport activities was allowed after 4–6 months after surgery.

All patients were evaluated postoperatively as follows: 1, 3 and 6 months and then yearly.

### Outcome measurements

Clinical assessment at baseline and at follow-up was performed by researchers that were blind to allocation. Baseline characteristics considered were as follows: age (years), gender, dominance, generalized ligamentous laxity (GLL) as measured with the Beighton score [2] (GLL was considered for Beighton score equal or greater than 4 [13]), duration of symptoms (months), age at first dislocation (years), number of dislocations, type of work, type of sport, sports activity level, labral injury pattern (Bankart or ALPSA lesion), and number of anchors and SLAP lesion (presence and type).

The primary outcome of the study was assessment of disability-related quality of life through the national validated version of the Disabilities of the Arm, Shoulder and Hand (DASH) questionnaire [12, 29]. This is a self-administered questionnaire that measures physical ability and symptoms of the upper extremity and explores the impact of functional impairment and pain on daily living tasks, as well as social and recreational activities, work, and sleep. The scoring system of the questionnaire is based on a metric scale, ranging from 0 points (minimum disability, best result) to 100 points (maximum disability, poorest result). Two optional modules were also administered for assessment of working capacity (Work-DASH) and ability to play sports or instruments (Sport-DASH).

Secondary outcomes included the evaluation of the shoulder function related to joint stability as measured by Rowe score [32]. It is an objective instrument composed of four items: shoulder function (score: 0–50), pain (score: 0–10), stability (score: 0–30) and range of movement (score: 0–10). Total score ranges from 0 (poor result) to 100 (excellent result).

Recurrence rate of dislocation was also considered a secondary outcome and defined as at least one episode of re-dislocation, while episodes of subluxation were recorded and scored within the Rowe scoring system [32]. Re-operation rate was also assessed; intraoperative and postoperative complications were recorded as well.

### Statistical analysis

Data analysis was performed with statistical software (SPSS v.25, IBM Inc., Harmonk, NY, USA). Normality of numerical data were assessed by Shapiro–Wilk test. Descriptive statistics were reported as means and standard deviations (SD) for normally distributed numerical data, otherwise medians and interquartile ranges (IQR) were used. Categorical data were expressed as frequencies and percentages.

Comparisons between groups both at baseline and at follow-up were performed by use of Student’s *t* test for normally distributed data, otherwise the Mann–Whitney *U*-test was used. Fisher’s exact test was used for analysis of categorical variables. Significance was set at *p* ≤ 0.05.

Sample size was calculated according to the primary outcome measurement (DASH score) and based on a previously published study [25]. On considering 10 points as the minimal clinically important difference (MCID) detectable with the DASH score [12], estimated sample size was 31 cases per group given α equal to 0.05 and power (1 – *β*) equal to 0.80. This value was increased to 39 per group to compensate for an eventual 20% maximum loss of patients at follow-up.

## Results

**Fig. 1:**
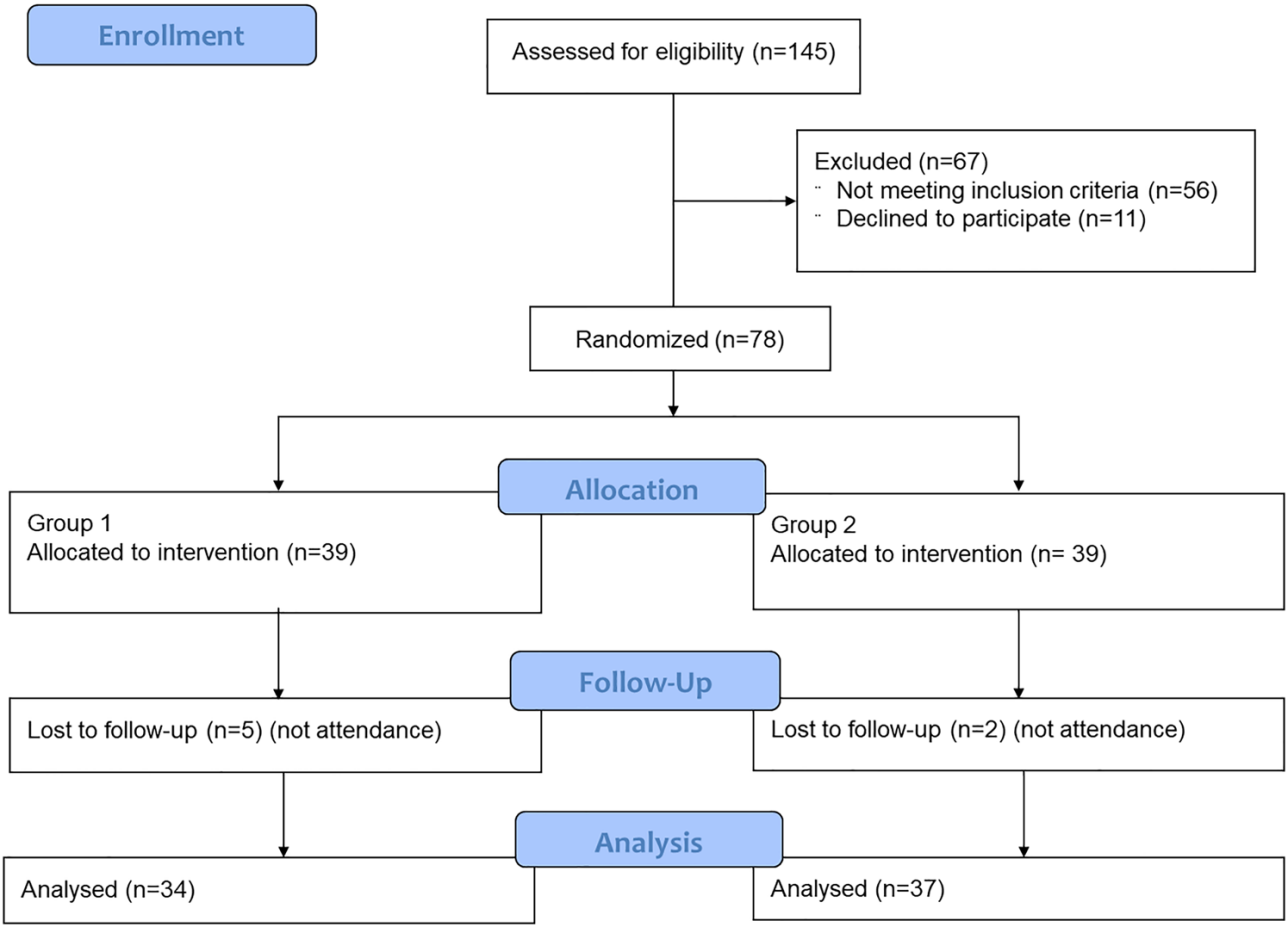
Consort flowchart of the study

Minimum follow-up was 3 years. Follow-up ranged from 36 to 60 months (median: 44; IQR: 13). Seven patients (9%) did not return at follow-up: five from group 1 and two from group 2. All these patients were called several times but refused to return because they lived in other regions of the country or abroad; Therefore, a total of 71 patients (91%) completed the follow-up: 34 from group 1 and 37 from group 2 (Fig. 1). Overall, there were 63 males (88.7%) and 8 females (8.3%). Age ranged from 18 to 47 years (median: 26; IQR: 13).

Baseline characteristics of the two groups are shown in Table 1. The two groups did not significantly differ with respect to all the independent variables.

**Table 1:** Comparison between treatment groups for baseline characteristics

Independent variables	Group 1 (*N* = 34)	Group 2 (*N* = 37)	*p* value
Age (years)			
Median (IQR)	24.5 (16.3)	27 (10.5)	n.s.
Gender			
Male, *N* (%)	30 (88.2%)	33 (89.2%)	n.s.
Female, *N* (%)	4 (11.8%)	4 (10.8%)	
Dominance			
*N* (%)	21 (61.8%)	22 (59.5%)	n.s.
Iperlaxity			
*N* (%)	9 (26.5%)	10 (27%)	n.s.
Age at first dislocation (years)			
Median (IQR)	18.5 (7.5)	22 (8.5)	n.s.
Time from first dislocation to surgery (months)			
Median (IQR)	35 (73)	40 (64.5)	n.s.
Type of work			
Manual, *N* (%)	4 (11.8%)	9 (24.3%)	n.s.
Sedentary, *N* (%)	30 (88.2%)	28 (75.7%)	
Type of sport			
None, *N* (%)	6 (17.6%)	2 (5.4%)	n.s.
Contact, *N* (%)	16 (47.1%)	26 (70.3%)	
Non contact, *N* (%)	12 (35.3%)	9 (24.3%)	
Overhead, *N* (%)	0	0	
Sports activity level			
None, *N* (%)	6 (17.6%)	2 (5.4%)	n.s.
Recreational, *N* (%)	14 (41.2%)	26 (70.3%)	
Competitive, *N* (%)	13 (38.2%)	9 (24.3%)	
Professional, *N* (%)	1 (2.9%)	0	
No. of dislocations			
Median (IQR)	7 (12)	4 (5.3)	n.s.
Injury pattern			
Bankart, *N* (%)	23 (67.6%)	20 (54.1%)	n.s.
ALPSA, *N* (%)	11 (32.4%)	17 (45.9%)	
SLAP lesion			
*N* (%)	5 (14.7%)	10 (27%)	n.s.
No. of anchors			
Median (IQR)	3 (1)	3 (2)	n.s.
Follow-up			
Median (IQR)	43.5 (9.3)	45 (14)	n.s.
DASH baseline			
Median (IQR)	23.9 (23.9)	22.7 (23.9)	n.s.
Work-DASH baseline			
Median (IQR)	9.4 (45.3)	18.8 (31.3)	n.s.
Sport-DASH baseline			
Median (IQR)	68.8 (40.6)	50 (46.9)	n.s.
Rowe score baseline			
Median (IQR)	35 (23.8)	35 (35)	n.s.

No significant differences between groups could be found for DASH score (Table 2), neither for working capacity and sports activity. Also, Rowe score was not significantly different in the two groups. Overall recurrence rate was 7%. Three re-dislocations were reported in group 1 and two in group 2 (n.s.). Only one patient in each group underwent re-operation (Table 2).

**Table 2:** Comparison between treatment groups at follow-up

Variables	Group 1 (*N* = 34)	Group 2 (*N* = 37)	*p *value
DASH			
Median (IQR)	4.6 (12.5)	2.3 (11.4)	n.s.
Work-DASH			
Median (IQR)	0 (0)	0 (0)	n.s.
Sport-DASH			
Median (IQR)	0 (9.4)	6.3 (25)	n.s.
Rowe score			
Median (IQR)	100 (22.5)	95 (20)	n.s.
Recurrence			
*N* (%)	3 (8.8%)	2 (5.4%)	n.s.
Revision surgery			
*N* (%)	1 (2.9%)	1 (2.7%)	n.s.

## Discussion

The main finding of the present paper is that there are no differences in clinical outcome of arthroscopic treatment of anterior shoulder instability between PEEK knotless and biodegradable knotted suture anchors at minimum 3-year follow-up. Moreover, no complications related to anchors material and design were reported.

The main strength of the present paper is that it allowed a comparison for two combined features: biodegradable versus permanent inert material, and knotless versus knotted anchor design. Indeed, literature is lacking and quite conflicting on both topics.

Several mechanical studies compared biodegradable and metal anchors for rotator cuff repair and showed contradictory results about differences in fixation strength of the implants [6, 7, 24]. Barber et al. [1] analyzed mechanical behavior of seven absorbable glenoid anchors and found no differences in ultimate failure strength after cyclic loading. Recently, Khoo et al. [14] compared four types of anchors used in labral repair. They found that biodegradable anchors were associated with less viable cells at 48 and 72 h after incubation and higher acidic culture medium at 24, 48, and 72 h, which basically means that examined biodegradable anchors are cytotoxic and have significantly lower failure load.

On the other side, no relevant biomechanical issues have been reported for PEEK anchors [10]. In a study focused on patellar tendon repair, Lanzi et al. [17] showed that PEEK anchors are superior to the transosseous technique for minimizing gap formation and improving load-to-failure strength, thus confirming the good mechanical properties of PEEK implants.

Moving forward to comparison between knotless and knotted anchors, a recent systematic review included five cadaveric studies focusing on arthroscopic Bankart repair [22]. The authors found contradictory results: one study claimed higher mechanical properties for knotless anchors [16], another favored knotted anchors [28], and the remaining three studies showed no differences between the two implant designs [20, 31, 33].

From a clinical standpoint, few studies try to clarify the controversies. Brown et al. [4] investigated surgical factors influencing the recurrence rate after arthroscopic Bankart repair. They included 26 studies (level of evidence varying from I to IV). Due to heterogeneity of included studies, a meta-analysis was not possible, but pooled weighted means showed that recurrent instability after arthroscopic Bankart repair was not influenced by rotator interval closure, number of anchors used, and not even by anchor material and design (i.e., biodegradable versus permanent anchors, and knotless versus knotted anchors). Although methodological quality of included studies was limited overall, results were consistent with those of the present study. Conversely, Peters et al. [30] conducted a prospective cohort study on 155 patients undergoing arthroscopic Bankart repair by comparing 4 types of knotless implants: biodegradable (PGA) tack, and biodegradable (PLLA), metal and PEEK suture anchors. The authors showed higher re-dislocation rate with the use of biodegradable implants. These results differed from those of the present study, probably because of the differences among groups for duration of follow-up (permanent anchors groups had shorter follow-up than biodegradable devices groups).

Recently, Matache et al. [22] conducted a systematic review comparing knotless and knotted anchors in patients undergoing arthroscopic Bankart repair. Four level II–III of evidence were included. Three studies found no significant differences in any clinical outcome measures [3, 15, 27], while one study found a significantly higher VAS and lower recurrence and revision rate with the use of knotted anchors [5]. However, meta-analysis showed no differences in revision rate between knotless and knotted anchors.

To summarize, although some mechanical studies found a certain “time zero” difference, clinical evidence showed that biodegradable and permanent anchors as well as knotless and knotted implants provide adequate outcomes, and this is confirmed in the present study. That being said, the main clinical advantage of knotless anchors, in our opinion, relies on their ease of use, which consistently reduces surgical times [22]. Regarding materials, as no adverse reactions have been reported in the literature with PEEK anchors, they should be preferred to biodegradable devices, which are burdened by a safety issue, as reported in the previous literature [8]. Based on these assumption, knotless PEEK anchors could be considered a viable alternative to knotted bioabsorbable anchors in clinical practice.

Main limitation of the present study is the absence of a postoperative imaging assessment. Therefore, no information could be provided about anchor resorption, peri-anchor cysts formation or osteoarthritis. Moreover, since the sample size was calculated according to the primary outcome measurement (DASH score), it is not possible to state if the sample could have been underrated to study eventual reactions related to anchor properties.

## Conclusions

The study showed no significant differences in clinical outcomes after arthroscopic treatment of traumatic anterior shoulder instability using PEEK knotless or biodegradable knotted anchors at minimum 3-year follow-up.
